# Supplementary material for: Improvement of Skin Condition Through RXR Alpha-Activating Materials
Source: Biomolecules. 2025 Feb 17;15(2):296. doi: 10.3390/biom15020296 (PMC11853381; doi:10.3390/biom15020296)
Supplement: Supplementary file 1 [file biomolecules-15-00296-s001.zip › biomolecules-3451998-supplementary.pdf]

Supplementary Material

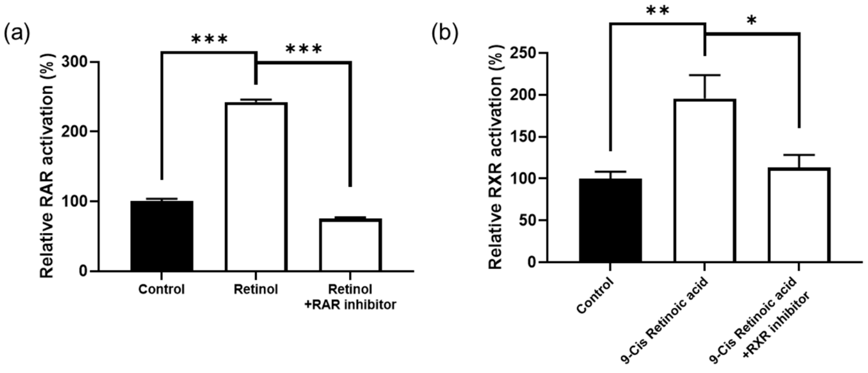

Figure S1. Inhibition efficacy of inhibitors on target receptors. **(a)** The effect of RAR inhibitor on inhibiting RAR activation. **(b)** The effect of RXR inhibitor on inhibiting RXR activation. Error bars indicate standard error of the mean. *$p < 0.05$, **$p < 0.01$, ***$p < 0.001$; One-way ANOVA analysis.

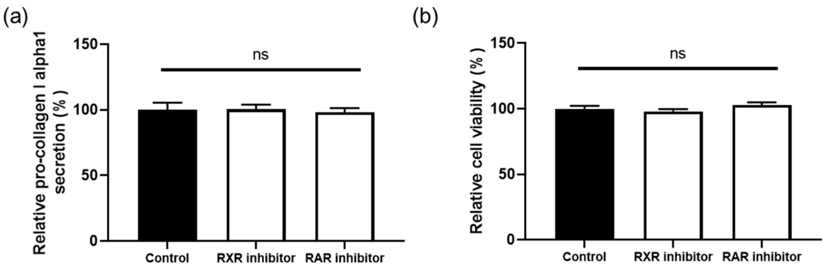

Figure S2. Evaluation of the potential side effects of inhibitors. **(a)** The effect of inhibitors on collagen synthesis **(b)** The effect of inhibitors on cellular viability. Error bars indicate standard error of the mean. One-way ANOVA analysis.
